# EGFR mediates hyperlipidemia-induced renal injury via regulating inflammation and oxidative stress: the detrimental role and mechanism of EGFR activation

**DOI:** 10.18632/oncotarget.8222

**Published:** 2016-03-21

**Authors:** Qilu Fang, Chunpeng Zou, Peng Zhong, Feng Lin, Weixin Li, Lintao Wang, Yali Zhang, Chao Zheng, Yi Wang, Xiaokun Li, Guang Liang

**Affiliations:** ^1^ Chemical Biology Research Center, School of Pharmaceutical Sciences, Wenzhou Medical University, Wenzhou, Zhejiang, China; ^2^ Department of Ultrasonography, the Second Affiliated Hospital, Wenzhou Medical University, Wenzhou, Zhejiang, China; ^3^ Department of Gynecology, the First Affiliated Hospital of Wenzhou Medical University, Wenzhou, Zhejiang, China; ^4^ Department of Endocrinology, the Second Affiliated Hospital, Wenzhou Medical University, Wenzhou, Zhejiang, China

**Keywords:** obesity, obesity-induced kidney injury, epidermal growth factor receptor, palmitate, c-Src

## Abstract

Previous studies have implicated inflammation, oxidative stress, and fibrosis as key factors in the development of obesity-induced kidney diseases. Epidermal growth factor receptor (EGFR) plays an important role in cancer development. Recently, the EGFR pathway has been increasingly implicated in chronic cardiovascular diseases via regulating inflammation and oxidative stress. However, it is unclear if EGFR is involved in obesity-related kidney injury. Using ApoE^−/−^ and C57BL/6 mice models and two specific EGFR inhibitors, we investigated the potential effects of EGFR inhibition in the treatment of obesity-related nephropathy and found that EGFR inhibition alleviates renal inflammation, oxidative stress and fibrosis. In NRK-52E cells, we also elucidated the mechanism behind hyperlipidemia-induced EGFR activation. We observed that c-Src and EGFR forms a complex, and following PA stimulation, it is the successive phosphorylation, not formation, of the c-Src/EGFR complex that results in the subsequent cascade activation. Second, we found that TLR4 regulates the activation EGFR pathway mainly through the phosphorylation of the c-Src/EGFR complex. These results demonstrate the detrimental role of EGFR in the pathogenesis of obesity-related nephropathy, provide a new understanding of the mechanism behind hyperlipidemia/FFA-induced EGFR activation, and support the use of EGFR inhibitors in the treatment of obesity-induced kidney diseases.

## INTRODUCTION

Obesity has become a major global health problem and has long been recognized as a risk factor for cardiovascular disease, glomerulopathy, and other chronic diseases [1, 2]. A number of studies have shown that hyperlipidemia can lead to glomerular structural and functional changes, and obesity has been linked as an independent risk factor for end stage renal disease [3, 4]. Elevated levels of free fatty acids (FFAs), such as palmitic acid (PA), in the bloodstream of obese patients have been shown to cause inflammation and oxidative stress in many of the body's organs, including the kidneys, resulting in increased insulin resistance, renal fibrosis, renal cell apoptosis and kidney lesions [5]. However, while there have been increased reports linking obesity to kidney injury, the underlying mechanisms behind how obesity significantly affects the progression and development of these disorders is still insufficiently understood.

The epidermal growth factor receptor(EGFR) pathway plays an important role in cell proliferation, differentiation and migration and has been extensively researched in its capacity in tumor progression [6]. Recently, the EGFR pathway has also been linked to the progression of other chronic diseases, such as such as insulin resistance, diabetic nephropathy and cardiomyopathy [7, 8]. EGFR inhibitors have been found to alleviate angiotensin 2-induced kidney disease, suppressing inflammation and oxidative stress [7, 9, 10]. These results suggest that the role of EGFR is not limited to just cell proliferation and fibrosis, but it can also function as a therapeutic target for chronic or metabolic diseases.

**Figure 1:**
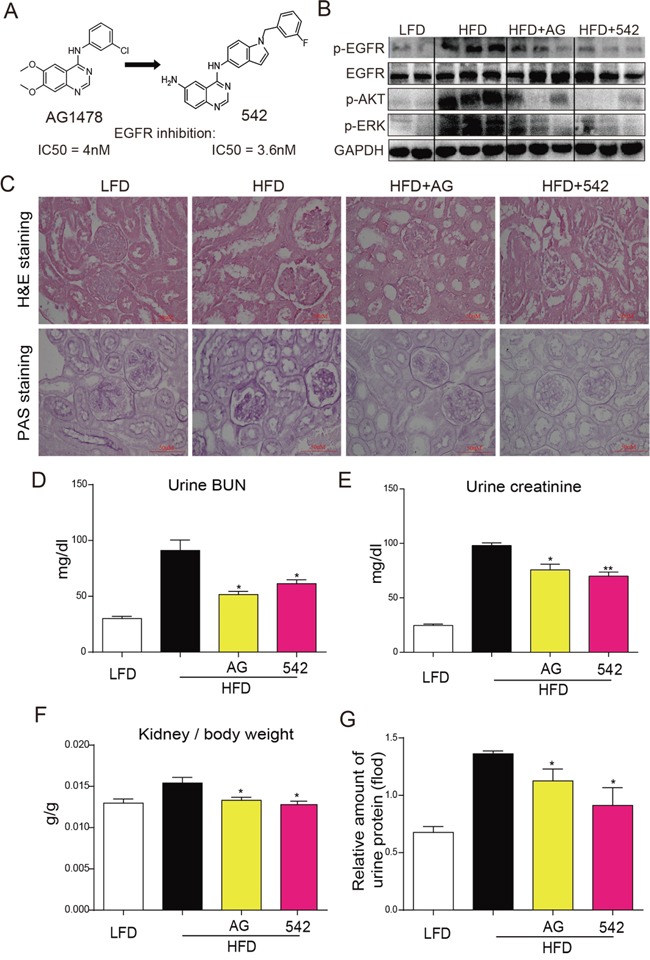
Oral administration of EGFR inhibitors suppressed HFD-induced EGFR signaling and attenuated kidney injury in ApoE^−/−^ mice **A.** Chemical structure of 542. **B.** Orally administered 542 significantly inhibited EGFR signaling, including phosphorylation of EGFR, AKT and ERK, in high fat diet (HFD)-fed ApoE^−/−^ mice.(Shown are representative western blots, n=2 in control group; n=3 in other three groups). **C-G.** 542 significantly improved structural changes and renal function in kidneys of obese mice. C. H&E staining was used for the analysis of histological abnormalities, PAS staining was used for the detection of glycogen (purple) in kidney section. D-G. BUN, creatinine, and urinary protein levels, as well as kidney/body weight ratio, were measured for the renal function test. Body weight and kidney weight of mice were recorded at the time of death. Data are means ± SEM (n=8 in four groups; ns, no significance; * *p*<0.05, ** *p*<0.01; vs. HFD group; LFD, low-fat diet; HFD, high-fat diet).

However, it is still unknown whether EGFR inhibition is able to alleviate the development of obesity-related disorders, such as hyperlipidemia-induced kidney diseases. This study aims to demonstrate the potential role and mechanism of EGFR in the pathogenesis of obesity-related kidney injury. In this study, EGFR was pharmacologically inhibited by two previously reported small-molecule inhibitors, AG1478 and 542 (Figure 1A) [11] in animal and cellular models, or genetically silenced by siRNAin renal cells. We foundthat EGFR inhibition diminished renal injury by reductionof inflammation, oxidative stress, apoptosis and fibrosis both *in vivo* and *in vitro* and that our results provided further support tounderstand the detrimental role and mechanism of EGFR activation in obesity-related kidney diseases.

## RESULTS

### Oral administration of EGFR inhibitors to HFD-fed ApoE^−/−^ mice suppressed EGFR signaling and attenuated kidney injury

Serum analysis for LDL and total cholesterol (TCH) at the conclusion of the experiment revealed that HFD significantly induced increases in serum LDL (Figure S1A) and TCH (Figure S1B) levels when compared to the LFD group. However, treatment with AG and 542 slightly reduced these HFD-induced increases. As shown in Figure 1B, 542 and AG administration suppressed EGFR phosphorylation as well as the downstream AKT and ERK activation in HFD-fed mouse kidney. H&E staining revealed an increase in glomerular shrinkage and kidney mesangial matrix expansion, while PAS staining revealed an increased accumulation of glycogen in the renal tissues of HFD-fed mice. Treatment with AG and 542 reversed such changes (Figure 1C). When examining the key biochemical indices for renal health and function, we observed that treatment with AG and 542 suppressed HFD-induced increases in BUN, creatinine, kidney/body weight ratio, and urine protein levels in HFD-fed ApoE^−/−^ mice (Figure 1D-1G). These results indicated that inhibition of EGFR can alleviate HFD-induced kidney injury.

### EGFR inhibitors improved HFD-induced fibrosis and apoptosis in the kidney tissues of HFD-fed ApoE^−/−^ mice

**Figure 2:**
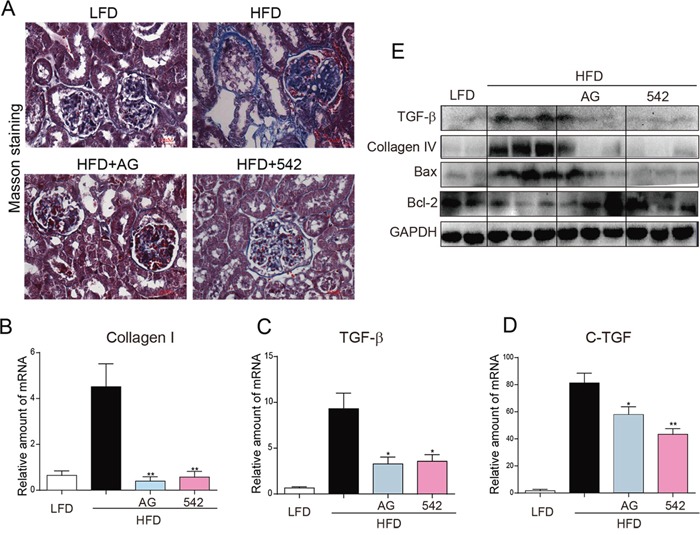
Oral administration of EGFR inhibitors improved HFD-induced fibrosis in the kidneys of ApoE^−/−^ mice **A.** Representative images of histological abnormalities in obese renal tissue. (400X). Masson staining was used for the detection of fibrosis (blue, respectively) in the kidney sections. **B-D.** Administration of 542 reduced the gene expression of the indicated genes related to fibrosis in the kidney tissue of obese mice. Renal tissues from each group were individually processed for mRNA extraction and RT-qPCR analysis. The mRNA levels of Collagen1 (B), TGF-β (C) and CTGF (D) were normalized by β-actin (n=5-7; ns, no significance; * *p*<0.05, ** *p*<0.01; vs. HFD group). **E.** Administration of 542 reduced the production of fibrotic cytokines and pro-apoptotic protein Bax, while increasing the expression of anti-apoptotic protein Bcl-2. Shown are representative western blots, n=2 in control group; n=3 in other three groups. (LFD, low-fat diet; HFD, high-fat diet)

As shown in Figure 2A, Masson staining revealed increased fibrosis (blue) in the kidney tissues of HFD-fed ApoE^−/−^ mice. However, these changes were significantly reduced in the 542-treated and AG-treated mice. Real-time qPCR assay showed significant increases in mRNA levels of pro-fibrotic genes Collagen-1, TGF-β and CTGF (Figure 2B-2D), and Western blot analysis revealed increases in the protein levels of TGF-β and Collagen-4 in HFD-fed mice (Figure 2E). In contrast, treatment with 542 and AG mitigated HFD-induced expression of these fibrotic genes. In addition, we observed that while HFD increased Bax expression and decreased Bcl-2 protein expression, both 542 and AG were able to reverse these changes (Figure 2E). These results suggest that EGFR inhibition can attenuate HFD-induced renal fibrosis and apoptosis.

### EGFR inhibitors attenuated HFD-induced inflammation and oxidative stress in the kidneys of HFD-fed ApoE^−/−^ mice

**Figure 3:**
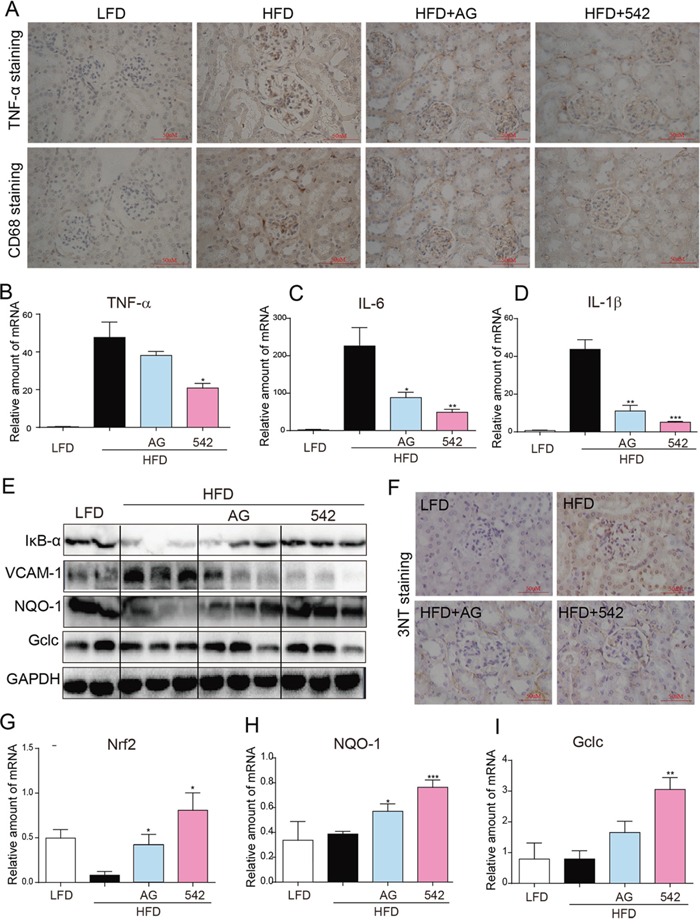
Oral administration of EGFR inhibitors reduced HFD-induced inflammation and ROS production in the kidneys of ApoE^−/−^ mice **A.** The administration of 542 for 2 months significantly reduced HFD-induced increases of kidney inflammation, including expression of TNF-α, and macrophage infiltration as characterized by CD68 staining. **B-D.** 542 also reduced the mRNA expression of TNF-α (B) and other inflammatory cytokines, such as IL-6 (C) and IL-1β (D). **E.** Administration of 542 also inhibited the degradation of IκB and expression of adhesion factors VCAM-1. **F-I.** 542 administration reduced the HFD-induced production of reactive oxygen species (ROS). Immunohistochemistry revealed that 542 decreased HFD-induced 3-NT (F) and RT-qPCR showed 542 increased mRNA levels of antioxidants, such as Nrf2 (G), NQO-1 (H) and Gclc (I), as well as the protein levels of antioxidant proteins NQO-1 and Gclc (E). (n=7/8; *p<0.05, **p<0.01, ***p<0.001; vs. HFD group; LFD, low-fat diet; HFD, high-fat diet)

Immunohistochemical staining revealed enhanced levels of TNF-α and CD68 in the renal tissues of HFD-fed ApoE^−/−^mice, when compared to LFD-fed mice (Figure 3A). The kidneys of HFD-fed ApoE^−/−^ mice also showed elevated mRNA expression of inflammatory cytokines, including TNF-α, IL-6, and IL-1β (Figure 3B-3D), reduced protein levels of IκB, and increased protein expression of cell adhesion molecule, VCAM-1 (Figure 3E). Oral administration of AG and 542 significantly reversed such changes in the renal tissues (Figure 3A-3E). In HFD-fed ApoE^−/−^ mice, we also observed an increased oxidative stress through the accumulation of 3-NT in the kidney tissues of HFD-fed mice (Figure 3F). In contrast, oral administration with AG and 542 attenuated HFD-induced oxidative stress, decreasing 3-NT accumulation, accompanied with increased mRNA expression of anti-oxidant genes Nrf2, NQO-1 and Gclc (Figure 3F-3I). Therefore, EGFR inhibition can mitigate both HFD-induced inflammation and oxidative stress. It was also observed that at the same dosage dose of 10 mg/kg, 542 showed stronger anti-inflammatory and antioxidant effects that AG.

### Oral administration of EGFR inhibitors to HFD-fed mice reduced serum LDL, TCH and TG levels, and attenuated kidney injury and fibrosis

As shown in Figure S2, HFD-fed mice exhibited increased body weight (A) and serum glucose (B), TCH (C), total triglyceride (TG; D) and LDL (E) levels. With the exception of serum glucose levels, these increases were ameliorated following treatment with AG and 542 at different degree. Furthermore, oral administration of AG and 542 improved HFD-induced increases in both serum albumin levels and kidney/body weight ratio (Figure S3A). Western blot analysis for kidney tissues revealed that AG and especially 542 significantly suppressed HFD-induced phosphorylation of EGFR, AKT and ERK (Figure S3B), and improved histological abnormalities and fibrosis in the renal tissues of obese mice (Figure S4). Further mRNA and protein analysis confirmed these observations, as AG and 542 were also able to markedly suppress HFD-induced mRNA expression of TGF-β (Figure S3C), Collagen-1 (Figure S3D), CTGF (Figure S4E), and protein expression of TGF-β and Collagen-4 (Figure S4F). In addition, western blot analysis of apoptosis regulator Bax and anti-apoptotic protein Bcl-2 also indicated that AG and 542 reduced apoptosis in HFD-fed mouse kidney (Figure S4F). Again, it was demonstrated that EGFR inhibitors could effectively improve HFD-induced kidney injury, but this time in a C57BL/6 mouse model.

### Oral administration of EGFR inhibitors mitigated HFD-induced kidney injury through inhibition of inflammation, oxidative stress and apoptosis in male C57BL/6 mice

**Figure 4:**
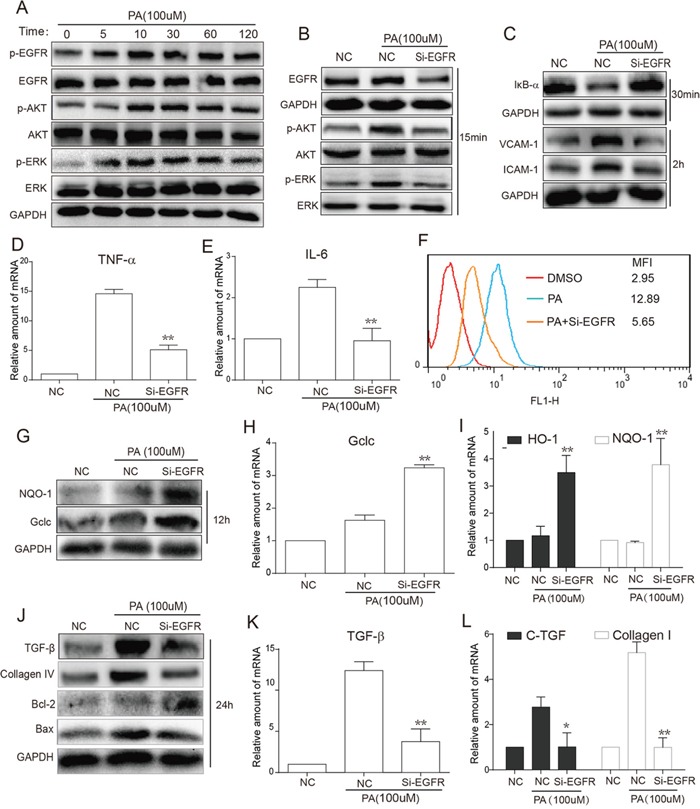
EGFR silencing inhibited PA-induced activation of EGFR signaling, inflammation, oxidation and fibrosis in NRK-52E cells **A.** PA-stimulated time-dependent activation of EGFR signaling. NRK-52E cells were stimulated with PA (100uM) for different various time periods (5, 15, 30, 60 and 120 min). Western blot analysis was then used to examine EGFR activation, including EGFR, AKT and ERK phosphorylation. **B.** Following PA stimulation for 15 min, total proteins were extracted from the cell lysate, and the phosphorylation levels of EGFR, AKT and ERK were examined by Western blot analysis using GAPDH as a loading control. **C.** Following PA stimulation for 30 min, the total proteins were extracted from the cell lysate, and the level of IκBα were examined by Western blot analysis with GAPDH as a loading control. After PA stimulation for 2 h, the levels of adhesion factors VCAM-1 and ICAM-1 were examined also by Western blot with GAPDH as a loading control. **F.** NRK-52E cells were pre-treated with Si-EGFR for 24h to block the EGFR gene and then stimulated with PA for 6h. Flow Cytometry was used for detecting the ROS. **G.** After PA stimulation for 12 h, antioxidant proteins NQO-1 and Gclc were examined by Western blot with GAPDH as a loading control. **J.** After PA stimulation for 24 h, fibrotic factors TGF-β and Collagen4, pro-apoptotic protein Bax and anti-apoptotic protein Bcl-2 were examined by Western blot with GAPDH as a loading control. **D-E, H-I, K-L.** NRK-52E cells were pre-treated with Si-EGFR for 24h to block the EGFR gene and then stimulated with PA for 24h. Total RNAs were extracted, and the mRNA levels of inflammatory, oxidant and fibrotic cytokines, such as TNF-α (D), IL-6 (E), Gclc (G), HO-1 and NQO-1 (H), TGF-β (J), CTGF and Collagen1 (K), were detected by real-time qPCR. (*p<0.05, **p<0.01, ***p<0.001; vs. the PA Group).

An increased state of inflammation and oxidative stress was also observed in HFD-fed C57BL/6 mice. Immunohistochemical staining revealed an increased accumulation of TNF-α, CD68 and 3-NT in the renal tissue (Figure S5A-S5C). The mRNA expression of inflammatory cytokines, such as IL-1β, IL-6, and TNF-α (Figure S3E-S3G) and protein expression of VCAM-1 (Figure S5D) was enhanced in HFD-fed mouse kidneys. Such increases were significantly inhibited in AG- and 542-treated HFD mice. Also treatment with AG or 542 enhanced the expression of antioxidant genes including HO-1, Gclc, and NQO-1at both mRNA and protein levels (Figure 4H-4J and Figure S5D). It was again observed that 542 was generally more potent than AG in terms of its anti-inflammatory and antioxidant activity.

### EGFR silencing inhibited PA-induced activation of EGFR signaling, inflammation, oxidative stress and fibrosis in NRK-52E cells

The *in vivo* experiments demonstrate that hyperlipidemia causes EGFR activation and EGFR inhibition attenuates obesity-induced renal injury. Then we aimed to validate the role of EGFR at the cellular level. According to the preliminary experiments, the concentration of PA at 100μM was used in the following cellular experiments. Firstly, Western blot analysis showed that PA treatment for 5-120 min remarkably increased the phosphorylation of EGFR and downstream AKT and ERK in renal NRK-52E cells (Figure 4A). To exclude possible non-specific inhibition by the small-molecule inhibitors, NRK-52E cells were transfected with an EGFR siRNA and then exposed to PA for the indicated times. Figure 4B revealed that EGFR silencingremarkably inhibited the PA-induced activation of AKT/ERK in cells treated with PA for 15 min. Furthermore, we tested the effects of EGFR silencing on PA-induced inflammation, oxidative stress, fibrosis, and apoptosis. In NRK-52E cells treated with PA for 30 min, EGFR silencing inhibited PA-induced IκBα degradation and protein expression of cell adhesion molecules VCAM-1 and ICAM-1 (Figure 4C). Real-time qPCR assay revealed that EGFR silencing suppressed mRNA expression of inflammatory cytokines TNF-α and IL-6 (Figure 4D-4E). Similar results were observed in ROS production and anti-oxidative gene expression. Through flow cytometry analysis of NRK-52E cells pre-treated with Si-EGFR for 24 h prior to 6 h PA stimulation, we saw that EGFR silencing significantly reduced PA-stimulated ROS production (Figure 4F). These findings were mimicked in the results of both Western blot analysis (12 h PA stimulation) for protein levels of NQO-1 and Gclc (Figure 4G) and real-time qPCR analysis (12 h PA stimulation) for mRNA levels of Gclc, HO-1 and NQO-1 (Figure 4H-4I), which revealed that EGFR silencing increased both protein and mRNA expression of these antioxidants. Furthermore, after PA stimulation for 24 h, we also observed that EGFR silencing also inhibited PA-increased protein levels of fibrotic factors, TGF-β and Collagen-4 (Figure 4J), and mRNA levels of TGF-β (Figure 4K), CTGF and Collagen-1 (Figure 4L). Similar results were also observed in the levels of apoptotic proteins Bax and Bcl2, indicating that EGFR knockdown attenuated PA-induced NRK-52Ecell apoptosis (Figure 4J).

### PA induced phosphorylation of EGFR via TLR4/c-Src signaling pathway in NRK-52E cells

**Figure 5:**
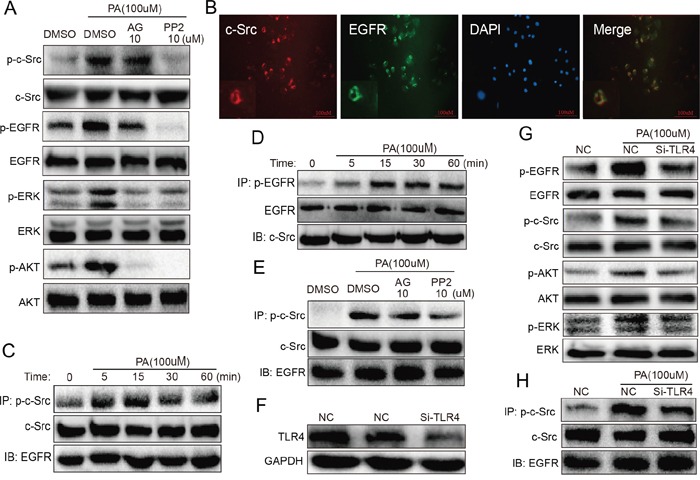
TLR4 regulated PA-induced phosphorylation of EGFR and c-Src in the EGFR/c-Src complex in NRK-52E cells (TLR4 silencing inhibits PA-induced phosphorylation of c-Src in the EGFR/c-Src complex and EGFR signaling in NRK-52E cells) **A.** c-Src is upstream of EGFR. NRK-52E cells were pre-treated with AG (inhibitor of EGFR) and PP2 (inhibitor of c-Src) for 1 h and then stimulated with PA for 15 min. Using Western blot analysis, we detected the c-Src and EGFR phosphorylation. Both AG and PP2 suppressed EGFR phosphorylation, but while PP2 inhibited PA-induced c-Src phosphorylation, AG had no effect. **B.** Immunofluorescence revealed that EGFR binds with c-Src without any stimulation. **C-D.** In the c-Src/EGFR complex, PA stimulated the phosphorylation of c-Src and then EGFR. Co-immunoprecipitation showed that in the complex, PA induced the phosphorylation of EGFR first 5 min after PA stimulation and s-Src 15 min after stimulation. **E.** Through co-immunoprecipitation, we further revealed that PP2 inhibited the phosphorylation of c-Src, whereas AG had no effect on c-Src phosphorylation in the c-Src/EGFR complex. **F.** Western blot analysis showing the efficiency of using SiRNA to silence TLR4 in NRK-52E cells. **G.** Silencing of TLR4 blocked PA-induced activation of EGFR signaling, inhibiting EGFR, c-Src, AKT and ERK phosphorylation. **H.** Silencing of TLR4 also significantly blocked PA-induced phosphorylation of c-Src in the EGFR/c-Src complex.

Above data indicated EGFR mediates HFD/PA-induced renal injuries, however, it is still unclear how PA triggers EGFR activation. It has been reported that fatty acid directly activates Toll-like receptor 4 (TLR4), which can induce c-Src kinase activation [12]. In addition, previous studies demonstrated that c-Src was involved in mediating Angiotensin II-induced EGFR transactivation [13]. Thus we speculate that PA triggers EGFR signaling pathway through the TLR4/c-Src signaling cascade. NRK-52E cells were pre-treated with either EGFR inhibitor AG or c-Src inhibitor PP2 for 1 h before being stimulated with PA for 15 min. As shown in Figure 5A, PP2 not only inhibited c-Src phosphorylation, but it also suppressed the phosphorylation of EGFR and its downstream targets ERK and AKT. However, AG was only able to inhibit phosphorylation EGFR, ERK and AKT, and not c-Src. These results suggest that c-Src is upstream of EGFR. Interestingly, the overlapped immunofluorescence images revealed the possibility that c-Src and EGFR may form a complex with one another in the absence PA stimulation (Figure 5B). Using co-immunoprecipitation, the c-Src/EGFR complex was validated and it was observed that PA stimulation induced the phosphorylation of both c-Src and EGFR in the c-Src/EGFR complex (Figure 5C-5D). However, while c-Src was significantly phosphorylated 5 min after PA stimulation, it took fifteen minutes of PA stimulation for EGFR phosphorylation to become evident (Figure 5C-5D). This indicates that there exists a sequence in the phosphorylation and activation of the c-Src/EGFR complex, where c-Src phosphorylation precedes that of EGFR under PA stimulation. Furthermore, using EGFR inhibitor AG and c-Src inhibitor PP2, it was observed that two inhibitors could not affect the complex formation, and while PP2 inhibited the phosphorylation of c-Src, AG had no effect on c-Src phosphorylation in the complex (Figure 5E). These observations provide further evidence suggesting: 1) c-Src and EGFR forms a complex in NRK-52E cells; and 2) c-Srcphosphorylation exists upstream of EGFR activation.

TLR4 silencing via siRNA transfection was used to examine the role of TLR4 in PA-induced EGFR and c-Src activation. As shown through in Figure 5F, the use of siRNA efficiently silenced TLR4 in NRK-52E cells. Further Western blot analysis demonstrated that TLR4 silencing blocked PA-induced phosphorylation of EGFR, c-Src, ERK and AKT (Figure 5G). Figure 5H then showed that TLR4 silencing remarkably inhibited the phosphorylation of c-Src in the EGFR/c-Src complex in PA-stimulated NRK-52E cells. These results indicated that TLR4 mediated PA-induced the activation of c-Src and then EGFR in c-Src/EGFR complex.

### Downstream of EGFR, AKT may play a more important role than ERK in PA-induced changes in NRK-52E cells

**Figure 6:**
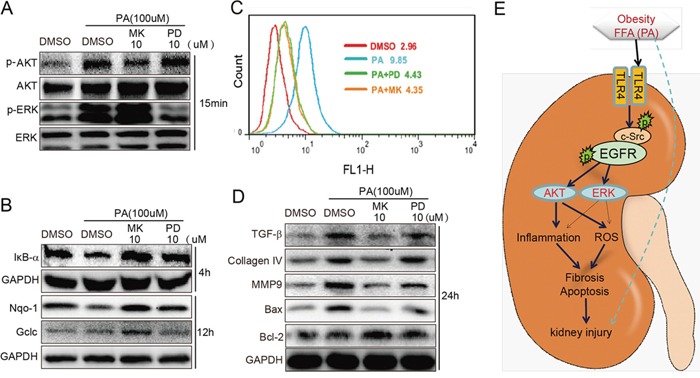
Inhibition of AKT and ERK attenuates PA-induced inflammation, ROS production, fibrosis and apoptosis in NRK-52E cells **A.** The efficiency of AKT inhibitor MK and ERK inhibitor PD when used the same concentration. **B-C.** MK and PD attenuated PA-induced inflammation and oxidation. MK and PD inhibited the degradation of IκB and production of ROS. MK and PD also increased the expression of antioxidant proteins NQO-1 and Gclc. **D.** MK and PD also inhibited PA-induced expression of fibrotic factors TGF-β, Collagen4, and MMP9 pro-apoptotic proteins Bax and anti-apoptotic protein Bcl-2. **E.** The proposed mechanism of TLR4/c-Src/EGFR signaling in obesity-related glomerulopathy (ORG).

AKT and ERK are the classical downstream pathways in EGFR signaling. In order to characterize the contribution of AKT and ERK in the PA-induced EGFR cascade, cells were treated with AKT inhibitor MK2206 (MK) and ERK inhibitor PD98059 (PD) at the same concentration of 10 μM before PA stimulation for 15 min. As shown in Figure 6A, MK was able to effectively block AKT phosphorylation, while PD was able to effectively block ERK phosphorylation. When we examined the impact of MK and PD on PA-induced inflammation and oxidative stress, shown in Figure 6B, we were able to see that both MK and PD inhibited the degradation of IκB and enhanced the expression of antioxidant proteins NQO-1 and Gclc. Flow cytometry also revealed that treatment with MK or PD reduced ROS production (Figure 6C). Moreover, Figure 6D showed that both MK and PD inhibited PA-induced expression of fibrotic factors TGF-β and Collagen-4 and pro-apoptotic protein Bax, and enhanced the expression of anti-apoptotic protein Bcl-2. Interestingly, it was also observed that in general, AKT inhibition was more effective than ERK inhibition. These results suggest that while both AKT and ERK are involved in PA-induced inflammation, oxidative stress, fibrosis and apoptosis, AKT may play a greater role in PA-induced changes in NRK-52E cells.

## DISCUSSION

Increased inflammation and oxidative stress due to obesity also contributes greatly to the development and progression of renal injury [14, 15]. While EGFR has been implicated as a therapeutic target for cancer therapy, EGFR pathway has also been linked to inflammation, oxidative stress, and fibrosis [16-18]. Nitta et al. presented the case that hyperactivity of EGFR increased oxidative stress, which is a key factor in the development of diabetes and related complications [19]. Chen et al. showed that EGFR-dependent ERK signaling mediated TGF-β expression in renal fibrosis. On the other hand, inhibition of EGFR signaling also reduced renal fibrosis by decreasing TGF-β dependent fibrogenesis [17]. Furthermore, Studies have suggested that EGFR signaling can promote renal and vascular fibrosis through a MAPK-mediated mechanism [20], and the overexpression of dominant negative EGFR or use of AG1478 (AG), an EGFR tyrosine kinase inhibitor, was observed to drastically reduce TGF-β and fibronectin expression in cultured cells [21]. Recently EGFR inhibitors have also been found to alleviate angiotensin 2-induced kidney disease [9], demonstrating that the EGFR pathway has a role in mitigating inflammation, oxidative stress and fibrosis, and opening the door to EGFR having a potential role in the progression obesity-related renal injury. Here, we observed that EGFR inhibitors, AG and 542, were able to reduce inflammation, oxidative stress, fibrosis and apoptosis *in vivo* in the kidneys of high fat diet-fed ApoE^−/−^ and C57BL/6 mice and *in vitro* in NRK-52E cells. We also observed improvements in as serum TCH, TG, LDL levels and even body weight (Figure S1 and S2). Thus, our observations demonstrated that EGFR inhibition could reduce hyperlipidemia levels, as well as inhibit hyperlipidemia-induced renal tissue injury. Further studies should focus on the mechanism by which EGFR inhibition decreases the blood lipid levels. These data validated the detrimental role of EGFR in the pathogenesis of obesity-related renal injury.

While the EGFR pathway and its role in inflammation and oxidative stress have become increasingly better understood, the mechanisms behind these observations are still lacking. Multiple studies have implicated the NF-κB pathway in obesity-related inflammation and oxidative stress [22-24]. The NF-κB pathway has also been linked to both TLR4 and EGFR family receptors. Aside from its role in the inflammatory response, the NF-κB pathway also plays an important role also in oxidative stress. Studies have demonstrated the presence of crosstalk between NF-κB and Nrf2 a basic leucine zipper protein that regulates the expression of antioxidant proteins and protects against injury or inflammation-induced oxidative stress [25, 26]. The EGFR signaling pathway has also been linked to Nrf2 through the PI3K-AKT pathway, which in turn regulates Nrf2 via the antioxidant response element (ARE) [27]. In this study, we provide further evidence linking EGFR activation to NF-κB activation. Furthermore, we saw that inhibition of AKT and ERK also prevented PA-induced degradation of IκB in NRK-52E (Figure 6B). These results indicate that NF-κB is an important mediator of EGFR signaling in PA-induced regulating inflammation and oxidative stress.

EGFR activation has been typically linked to unsaturated, not saturated, fatty acids (FAs). It has been demonstrated that only unsaturated FAs and not saturated FAs resulted in tyrosine phosphorylation of EGFR in human endothelial cells [28], and in an EGFR T17 cell line, it was unsaturated FAs that were responsible for disrupting cellular, transmembrane EGFR signaling [29], indicating the inability of SFAs to directly bind to EGFR. However, in the current study, PA, a saturated FA, induced the phosphorylation and activation of EGFR, as well as its downstream targets in NRK-52E cells, suggesting saturated fatty acids are still capable of indirectly activating EGFR phosphorylation and cascade.

Several studies have reported that saturated FAs can activate toll-like receptor 4 (TLR4), and saturated FA-induced effects are mediated by TLR4 activation [30-32]. We observed that phosphorylation of EGFR, as well as AKT and ERK, was drastically suppressed, confirming TLR4's role as an upstream regulatory protein of EGFR (Figure 5G). To further examine the mechanism behind how TLR4 regulates EGFR signaling, we examined the role of c-Src, which has been previously linked to TLR4 and EGFR activation [33-35]. We observed that PA activates the EGFR pathway through the TLR4/c-Src signaling pathway. The more important is that we discovered through immunofluorescence that c-Src is capable of forming a complex with EGFR, with or without stimulation (Figure 5B). Co-immunoprecipitation analysis further showed that in the c-Src/EGFR complex, c-Src is phosphorylated first and then EGFR (Figure 5C-5D), indicating there exists a sequence in EGFR activation and suggesting that it is phosphorylation of the c-Src/EGFR complex and not the formation of the complex that is critical to EGFR activation. This study in the first time discovered the c-Src/EGFR complex model for EGFR activation innon-malignant cells.

Compound 542 is a new analog of AG1478. We previously demonstrated that it could specifically target EGFR and block Ang II-induced renal fibrosis via inhibiting EGFR activation [11]. In this study, although AG can significantly reverse HFD-induced pathological changes, 542 is more potent than AG generally. We presume that 542 has stronger EGFR inhibitory ability and 542 has a better pharmacokinetic profile *in vivo* than AG after chemical structure modification. In addition, we noted that 542 can reduce serum lipid level in both ApoE−/− mice (Figure S1) and WT mice (Figure S2), while AG only showed slightly effects on hyperlipidemia. Reducing serum lipid level may contribute to the advantage of 542 over AG in the treatment of obesity-induced kidney injuries. Certainly, we can not exclude the off-targeting effects of 542 *in vivo* according to the differences between AG and 542. Therefore, only AG and silencing EGFR expression by siRNA were used for the validation of the role of EGFR in the cellular level.

In conclusion, the results from this experiment help to elucidate the mechanisms behind TLR4/c-Src/EGFR signaling in the pathogenesis of obesity-related renal injury. The proposed mechanism can be found in Figure 6E. Briefly, hyperlipidemia/FFAs activate TLR4, resulting in the phosphorylation of c-Src and then EGFR in c-Src/EGFR complex and subsequent activation of AKT and ERK. The effect of EGFR activation is the activation of the NF-κB pathway and the upregulation of key genes involved in the inflammatory response and oxidative stress, contributing to the pathogenesis of obesity-related renal injury. We also shed light on the understanding of how hyperlipidemia and saturated fatty acids can activate the EGFR signaling pathway through TLR4 and c-Src/EGFR complex. This study demonstrates that EGFR is deeply involved in the pathogenesis of obesity-related renal injury and provides strong evidence for targeting the EGFR pathway for the treatment of this disease.

## MATERIALS AND METHODS

### Reagents and cell culture

Majority of reagents, kits, small molecular inhibitors, and antibodies are described in online Supplementary File. Compound 542 was prepared by our lab with a HPLC purity of 99.4%. 542 and AG1478 were dissolved in DMSO for *in vitro* experiments and in sodium carboxyl methyl cellulose (CMC-Na) (1%) for *in vivo* experiments. Epithelial rat kidney-derived cell line NRK-52E was obtained from the Shanghai Institute of Biochemistry and Cell Biology (*Shanghai, China*) and cultured in DMEM medium Gibco(*Eggenstein, Germany*) containing 1 g/L glucose supplemented with 10% FBS, 100 U/ml of penicillin, and 100 mg/ml of streptomycin in a humidified atmosphere of 95% air and 5% CO_2_ at 37°C.

### Animals

Male C57BL/6 mice weighing 18–22 g were obtained from the Animal Centre of Wenzhou Medical University (*Wenzhou, China*). Male ApoE^−/−^ mice, weighing 18-20 g and aged 8 weeks, were purchased from Beijing HFK Bioscience Co., Ltd (*Beijing, China*). Animals were housed at a constant room temperature with a 12:12 h light–dark cycle and fed a standard rodent diet and water. The animals were acclimatized to the laboratory for at least 3 days before being used. All animal care and experimental procedures were approved by the Wenzhou Medical University Animal Policy and Welfare Committee (*Approval Document No. wydw2014-0117*).

### Real-time quantitative PCR and western blotting analysis

RT-qPCR assay and Western blot analysis from cells or heart tissues are described in the Online Supplementary File and the gene primers for RT-qPCR are listed in Online Table S1.

### Immunofluorescence for EGFR and c-Src

After treatment, NRK-52E cells were directly fixed with 4% paraformaldehyde for 10min, 1% BSA in PBS for 30 min. Cells were incubated overnight at 4°C with EGFR and c-Src antibody (1:200), and then incubated with fluorescein isothiocyanate (FITC or PE)-labeled secondary antibody (Santa Cruz; 1:300) for 1 h. The nucleus was incubated with DAPI for 5 min, and then the images were viewed under fluorescence microscope (200×, amplification; Nikon).

### Immunoprecipitation

Collected cells or samples were lysed. Lysates (300-500 μg) were added with EGFR or c-Src antibody overnight at 4°C. The protein A/G beads were added, and the lysate mixture was shaken in room temperature for 2 hours before being washed for 5 times with PBS. The supernatant was removed and added with 1× loading buffer for Western blot analysis.

### siRNA-induced gene silencing

Gene silencing was achieved using the siRNA technique. EGFR or TLR4 siRNA was purchased from Gene Pharma (*Shanghai, China*). Transfection of NRK-52E cells with siRNAs was carried out using LipofectAMINE™ 2000 (*Invitrogen, Carlsbad, California*), according to the manufacturer's instruction. The transfected cells were then treated with palmitate for the following experiments.

### Determination of ROS generation by flow cytometry

In order to analyze the ROS generation in cells, a subtype of ROS such as hydrogen peroxide (H_2_O_2_) was detected using 2 μM DCFH-DA, respectively, as described previously. The fluorescence intensity for 10,000 events was acquired using FACS.

### Animal experiments

#### ApoE^−/−^ obesity model

ApoE^−/−^ mice were fed with high-fat diet (ApoE-HFD) andthe control ApoE^−/−^ mice were fed with normal diet (low-fat diet, LFD, n=8) for 16weeks. Since 9^th^ week, the ApoE-HFD mice were then randomly divided into three groups: ApoE-HFD (n=8), AG-treated ApoE-HFD (ApoE-HFD+AG, n=8) and 542-treated ApoE-HFD (ApoE-HFD+542, n=8). In the treated groups, the mice were orally administered 10mg/kg/day of AG or 542 for 8 weeks.

#### Normal obesity model

The C57BL/6 wide-type mice were fed with high-fat diet (HFD) andthe control mice were fed with low-fatdiet (Ctrol, n=8) for 16weeks. Since 9^th^ week, the HFD mice were then randomly divided into three groups: HFD (n=8), AG-treated HFD (HFD+AG, n=8) and 542-treated HFD (HFD+542, n=8). In the treated groups, the mice were orally administered 10mg/kg/day of AG or 542 for 8 weeks.

Low-fat diet was purchased fromMediScience Diets Co. LTD, Yangzhou, China, containing 10 kcal.% fat, 20 kcal.% protein and 70 kcal.% carbohydrate (Cat. #MD12031); high-fat diet was purchased from the same company (Cat. #MD12033) containing 60 kcal.% fat, 20 kcal.% protein and 20 kcal.% carbohydrate. AG1478 or 542 were given daily by oral gavage at a dose of 10 mg/kg/day. Mice in the control LFD and HFD groups were gavaged with vehicle (1% CMC-Na solution) only. The HFD groups and control groups received just 1% CMC-Na solution according to the same schedule. Body weight was recorded weekly. At the end of experiments, animals were sacrificed under ether anesthesia, and the blood samples were collected at the time of death. Kidney tissues were also collected and either embedded in 4% paraformaldehyde for pathological analysis and/or snap-frozen in liquid nitrogen for gene and protein expression analysis.

### Histopathology and immunohistochemistry

The immunohistochemical staining from frozen or formalin-fixed heart tissues (H&E, PAS, Sirius Red, Masson, anti-CD68, anti-TNF-α, and anti-3-NT) is described in the Online Supplementary File. The stained sections were viewed under light microscope (200X or 400X magnification).

### Measurements of the level of serum lipid and biochemical indicators

The components of serum lipid including the total triglyceride (TG), low-density lipoprotein (LDL), and total cholesterol (TCH) were measured. The Function Index for the kidneys including ALB, BUN, creatinine and urinary protein were also detected using commercial kits (*NanjingJiancheng, Jiangsu, China*).

### Statistical analysis

Data were presented as means ± SEM. The statistical significance of differences between groups was obtained by the student's t-test or ANOVA multiple comparisons in GraphPad Pro (*GraphPad, San Diego, CA*). Differences were considered to be significant at *p*< 0.05.

## SUPPLEMENTARY DATA



## Abbreviations

EGFR: Epidermal growth factor receptor
HFD: high fat diet
PA: palmitic acid
FFAs: free fatty acids
LDL: low density lipoprotein
TCH: total cholesterol
TG: total triglyceride
