# Supplementary material for: EGFR mediates hyperlipidemia-induced renal injury via regulating inflammation and oxidative stress: the detrimental role and mechanism of EGFR activation
Source: Oncotarget. 2016 Mar 21;7(17):24361–73. doi: 10.18632/oncotarget.8222 (PMC5029707; doi:10.18632/oncotarget.8222)
Supplement: Supplementary file 1 [file oncotarget-07-24361-s001.pdf]

SUPPLEMENTARY DATA

Reagents and cell culture

Palmitate acid (PA) and AG1478 (AG) were purchased from Sigma-Aldrich (St. Louis, MO). Compound 542 was prepared by our lab with a HPLC purity of 99.4%. AG and 542 were dissolved in dimethyl sulfoxide (DMSO) for *in vitro* experiments and 1% sodium carboxymethyl cellulose (CMC-Na) for *in vivo* experiments. MK-2206 (AKT inhibitor) was purchased from Selleck Chemicals (*Shanghai, China*). PD-098059 (ERK inhibitor) was purchased from Beyotime Biotechnology (*Shanghai, China*). Antibodies for GAPDH, EGFR, p-EGFR and p-AKT were purchased from Cell Signaling (Danvers, MA, USA), and antibodies for IκBα, AKT, p-ERK, ERK, TGF-β, Collagen IV, Bax, Bcl-2, VCAM-1, NQO-1, CD68, ICAM-1, p-c-Src, c-Src and TLR4 were purchased from Santa Cruz Biotechnology Co., Ltd (Shanghai, China). Antibodies for GCLC and 3-NT were purchased from Abcam (*Shanghai, China*). NRK-52E epithelial rat kidney-derived cell line was obtained from the Shanghai Institute of Biochemistry and Cell Biology (*Shanghai, China*) and cultured in DMEM medium Gibco (*Eggenstein, Germany*) containing 1 g/L glucose supplemented with 10% FBS, 100 U/ml of penicillin, and 100 mg/ml of streptomycin in a humidified atmosphere of 95% air and 5% CO_2 at 37°C.

Animals

Male C57BL/6 mice weighing 18–22 g were obtained from the Animal Centre of Wenzhou Medical University (*Wenzhou, China*). Male $ApoE^{-/-}$ mice, weighing 18-20 g and aged 8 weeks, were purchased from Beijing HFK Bioscience Co., Ltd (*Beijing, China*). Animals were housed at a constant room temperature with a 12:12 h light–dark cycle and fed a standard rodent diet and water. The animals were acclimatized to the laboratory for at least 3 days before being used. All animal care and experimental procedures were approved by the Wenzhou Medical University Animal Policy and Welfare Committee (*Approval Document No. wydw2014-0117*).

Real-time quantitative PCR

Cells or kidney tissues (50–100 mg) were homogenized in TRIzol (*Invitrogen, Carlsbad, CA*) for extraction of RNA according to the manufacturer's protocol. Both reverse transcription and quantitative PCR were carried out using a two-step M-MLV Platinum SYBR Green qPCR SuperMix-UDG kit (*Invitrogen, Carlsbad, CA*). Eppendorf's Mastercycler ep realplex detection system (*Eppendorf, Hamburg, Germany*) was used for q-PCR analysis. The primers of genes, listed in Table S1, were obtained from Invitrogen (*Shanghai, China*). The amount of each gene was determined and normalized to the amount of β-actin.

Western immunoblot analysis

Collected cells or homogenized kidney tissue samples were lysed. Lysates (60-100 µg) were separated by 10% SDS-PAGE and electrotransferred onto a nitrocellulose membrane. Each membrane was pre-incubated for 1.5 h at room temperature in Tris-buffered saline, pH 7.6, containing 0.05% Tween 20 and 5% non-fat milk. Then, the membrane was incubated with specific antibodies. Immunoreactive bands were detected by incubating the membrane with a secondary antibody conjugated with horseradish peroxidase and visualized using enhanced chemiluminescence reagents (*Bio-Rad, Hercules, CA*). The protein levels were analyzed using Image J analysis software version 1.38e and normalized to their respective controls.

Immunofluorescence for EGFR and c-Src

After treatment, NRK-52E cells were directly fixed with 4% paraformaldehyde for 10min, 1% BSA in PBS for 30 min. Cells were incubated overnight at 4°C with EGFR and c-Src antibody (1:200), and then incubated with fluorescein isothiocyanate (FITC or PE)-labeled secondary antibody (Santa Cruz; 1:300) for 1 h. The nucleus was incubated with DAPI for 5 min, and then the images were viewed under fluorescence microscope (200×, amplification; Nikon).

Immunoprecipitation

Collected cells or samples were lysed. Lysates (300-500 µg) were added with EGFR or c-Src antibody overnight at 4°C. The protein A/G beads were added, and the lysate mixture was shaken in room temperature for 2 hours before being washed for 5 times with PBS. The supernatant was removed and added with 1× loading buffer for Western blot analysis.

Animal experiments

$ApoE^{-/-}$ obesity model

To induced hyperlipidemia, $ApoE^{-/-}$ mice were purchased from HFK Bioscience and fed with high-fat diet (HFD). In contrast, the control $ApoE^{-/-}$ mice were fed with normal diet (low-fat diet, LHD) for 2 months. The HFD-fed mice were then randomly divided into three groups: HFD (n=8), AG-treated HFD (HFD+AG, n=8) and

542-treated HFD (HFD+542, n=8). In the HFD+AG group and HFD+542 group, the mice were orally administered 10mg/kg/day of AG or 542. The HFD group and age-matched control group (n=8) received just 1% CMC-Na solution according to the same schedule. Body weight was recorded weekly after AG or 542 administration. After 8-week treatment, animals were sacrificed under ether anesthesia, and the blood samples were collected at the time of death. Kidney tissues were also collected and either embedded in 4% paraformaldehyde for pathological analysis and/or snap-frozen in liquid nitrogen for gene and protein expression analysis.

Normal obesity model

Male C57BL/6 mice weighing 18–22 g were obtained from the Animal Centre of Wenzhou Medical University (Wenzhou, China). After an acclimatization period of one week, the C57BL/6 mice HFD group were fed with high-fat diet, while the control group fed with normal diet. After 2 months, the HFD-fed mice were then randomly divided into three groups: HFD (n=8), AG-treated HFD (HFD+AG, n=8) and 542-treated HFD (HFD+542, n=8). In the HFD+AG group and HFD+542 group, the mice were orally administered 10mg/kg/day of AG or 542. The HFD group and age-matched control group (n=8) received just 1% CMC-Na solution according to the same schedule. Body weight was recorded weekly after AG/542 administration. After 8-week treatment, animals were sacrificed under ether anesthesia, and the blood samples were collected at the time of death. Kidney tissues were also collected and either embedded in 4% paraformaldehyde for pathological analysis and/or snap-frozen in liquid nitrogen for gene and protein expression analysis.

Pathological analysis

Kidneys from C57BL/6 mice and ApoE$^{-/-}$ mice were fixed in 4% paraformaldehyde and embedded in paraffin. The paraffin sections (5 μm) were stained with hematoxylin and eosin (H&E) for histopathological analysis, 0.5% periodic acid and Schiff solution (PAS) for glycogen accumulation, 0.1% Sirius Red for type IV collagen accumulation, and Masson's trichrome stain for fibrosis. The specimens were observed under a light microscope (200× or 400×; *Nikon, Tokyo, Japan*).

Immunohistochemistry

After deparaffinization and rehydration, 5-μm kidney or heart sections (fixed on slides) were treated with 3% H_2O_2 for 10 min and with 1% bovine serum albumin in phosphate-buffered saline (PBS) for 30 min. Slides were incubated overnight at 4°C with anti-CD68, anti-TNF-α and anti-3-NT antibody (1:200) and then incubated with horseradish peroxidase-conjugated secondary antibody (*Santa Cruz Biotechnology, Santa Cruz, CA, USA*; 1:500) for 1 h at room temperature. The slides were subsequently dehydrated with graded alcohol and xylene and mounted with neutral resins. The specimens were observed under a light microscope (400×; *Nikon, Tokyo, Japan*).

siRNA-induced gene silencing

Gene silencing was achieved using the siRNA technique. EGFR or TLR4 siRNA was purchased from Gene Pharma (*Shanghai, China*). Transfection of NRK-52E cells with siRNAs was carried out using LipofectAMINE ™ 2000 (*Invitrogen, Carlsbad, California*), according to the manufacturer's instruction. The transfected cells were then treated with palmitate for the following experiments.

Measurements of the level of serum lipid and biochemical indicators

The components of serum lipid including the total triglyceride (TG), low-density lipoprotein (LDL), and total cholesterol (TCH) were measured. The Function Index for the kidneys including ALB, BUN, creatinine and urinary protein were also detected using commercial kits (*Nanjing Jiancheng, Jiangsu, China*).

Determination of ROS generation by flow cytometry

In order to analyze the ROS generation, a subtype of ROS such as hydrogen peroxide (H_2O_2) was detected using 2 μM DCFH-DA, respectively, as described previously. The fluorescence intensity for 10,000 events was acquired using FACS.

Statistical analysis

Data were presented as means ± SEM. The statistical significance of differences between groups was obtained by the student's t-test or ANOVA multiple comparisons in GraphPad Pro (*GraphPad, San Diego, CA*). Differences were considered to be significant at $p < 0.05$.

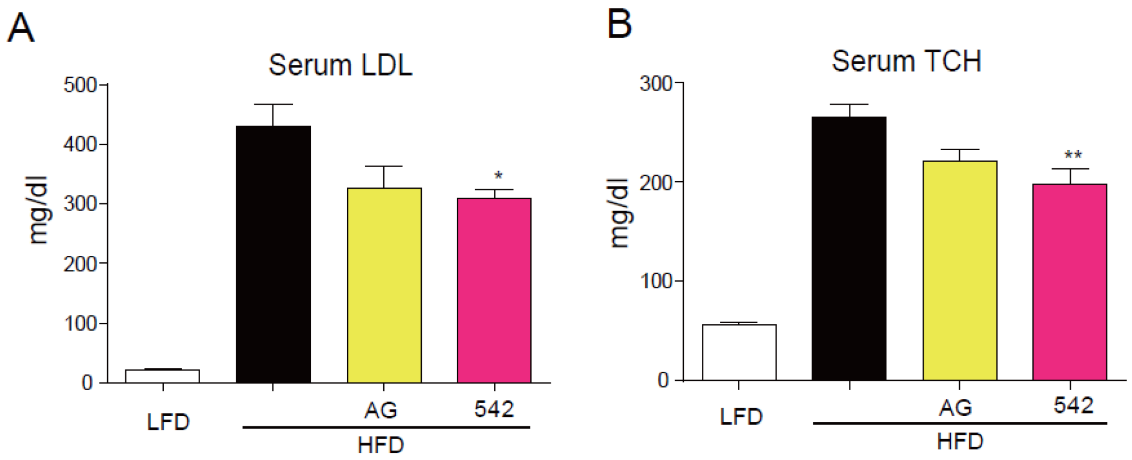

Figure S1: EGFR inhibition reduced serum levels of LDL and TCH in HFD-fed ApoE$^{-/-}$ mice. ApoE$^{-/-}$ mice were fed with low-fat diet (LFD) or high-fat diet (HFD), respectively, for 12 weeks, and pre-treated with AG (AG, 10 mg/kg) or 542 (10 mg/kg) for 8 Weeks. Serum LDL **A.** and TCH **B.** were detected using their respective kits. As shown in the figures, 542 obviously decreased the HFD-induced increases in LDL and TCH levels in ApoE$^{-/-}$ mice. (n=8; * $p<0.05$, ** $p<0.01$; vs. HFD group).

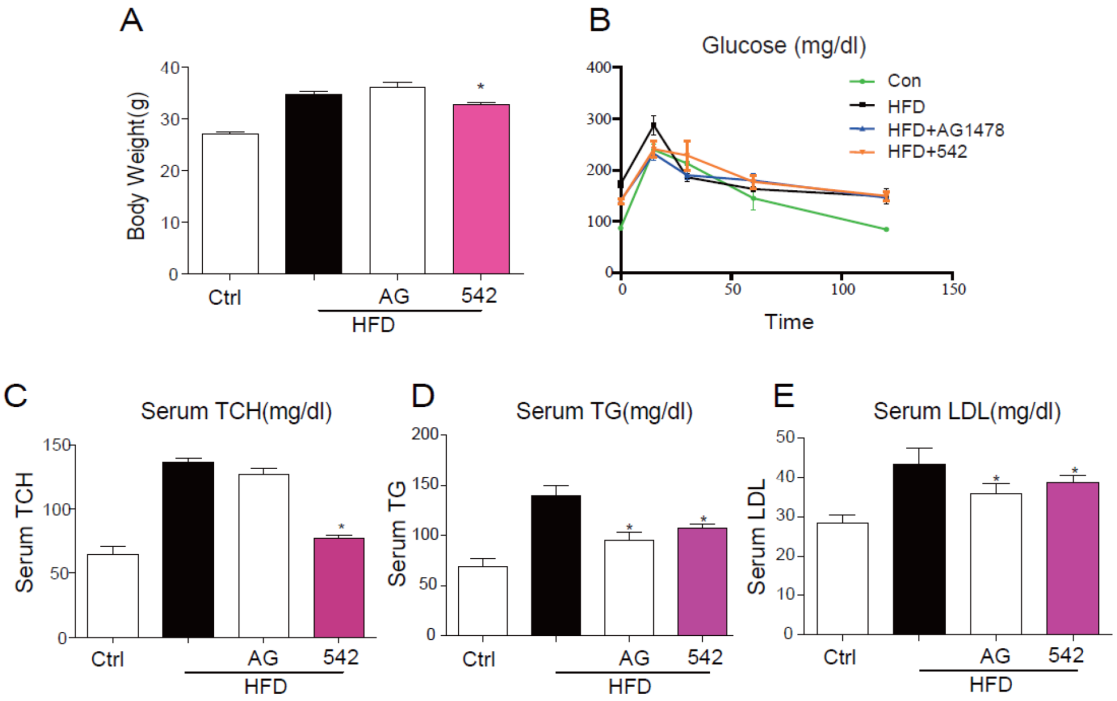

Figure S2: EGFR inhibition reduced serum levels of TCH, TG, and LDL, as well as body weight in C57BL/6 mice. A-B. Administration of 542 decreased the HFD-induced body weight gain (A), but had no effect on the serum glucose levels (B). **C-E.** 542 also significantly inhibited HFD-induced increases in TCH (C), TG (D) and LDL (E) levels in HFD-fed mice. (n=8; * p<0.05; vs. HFD group)

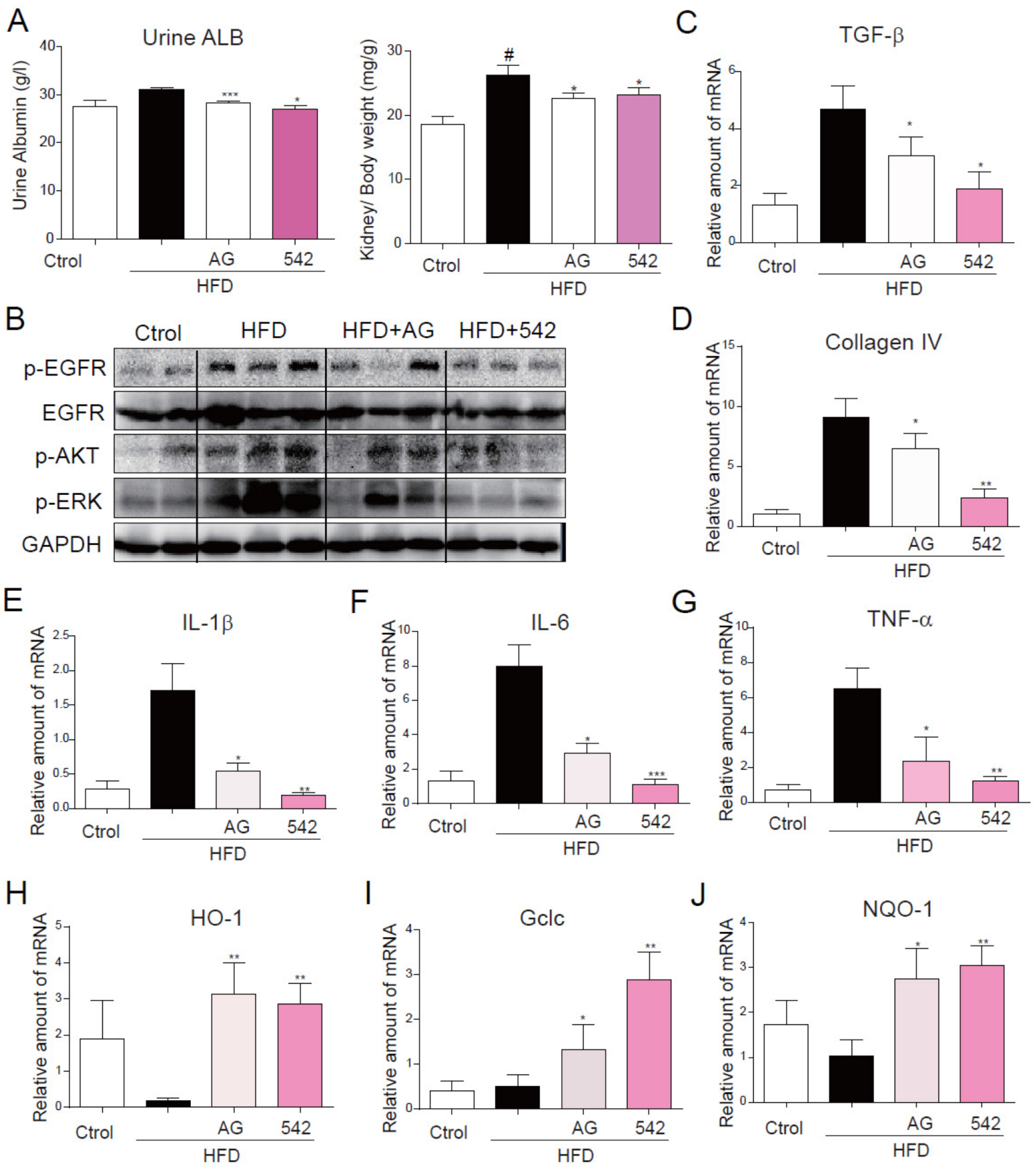

Figure S3: Oral administration of EGFR inhibitors attenuated kidney injury through inhibition of HFD-induced inflammation and ROS production in C57BL/6 mice. A. 542 improved HFD-induced increases in Urine ALB levels and kidney/body weight ratio. **B.** Tissues were homogenized and processed for Western blot analysis for EGFR signaling, which revealed that treatment with 542 significantly inhibited HFD-induced phosphorylation of EGFR, AKT and ERK. **C-J.** Tissues were homogenized in TRIzol for RT-qPCR. Administration with 542 decreased the expression of fibrotic factors TGF-β (C) and CTGF (D), as well as the expression of inflammatory cytokines IL-1β (E), IL-6 (F), and TNF-α (G). 542 also enhanced the expression of antioxidant proteins HO-1 (H), Gclc (I), and NQO-1 (J). (n=1/8; *p<0.05, **p<0.01, ***p<0.001; vs. HFD group).

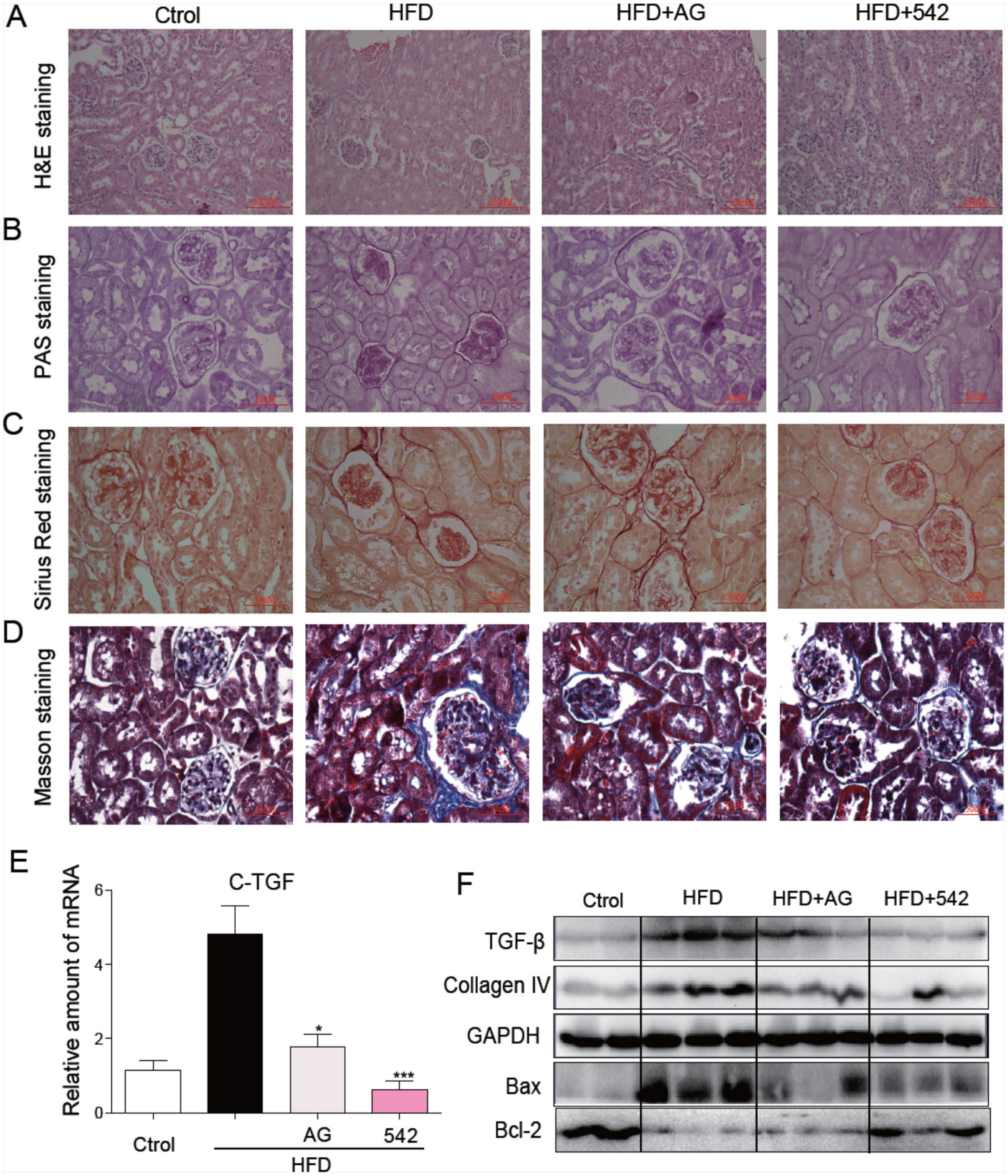

Figure S4: EGFR inhibition attenuates HFD-induced fibrosis and apoptosis in the kidneys of C57BL/6 mice. **A-D.** Representative images of histological abnormalities in diabetic renal tissues (200× or 400×) are shown. 542 significantly improved the pathological changes in the kidneys of obese mice. H&E staining (A) was used for analysis of histological abnormalities; PAS (B) was used for the detection of glycogen (purple); Masson staining (blue; C) and Sirius red staining (red; D) used for the detection of fibrosis in the kidney sections. **E.** Renal tissues from each group were individually processed for mRNA extraction and RT-qPCR analysis. The mRNA levels of CTGF were normalized to β-actin (n=5-7; *p<0.05, **p<0.01, ***p<0.001; vs. HFD group). **F.** Western blot analysis revealed that 542 administration reduced the production of fibrotic cytokines TGF-β and Collagen4, as well as the expression of pro-apoptotic protein Bax and anti-apoptotic protein Bcl-2. GAPDH was used as a loading control.

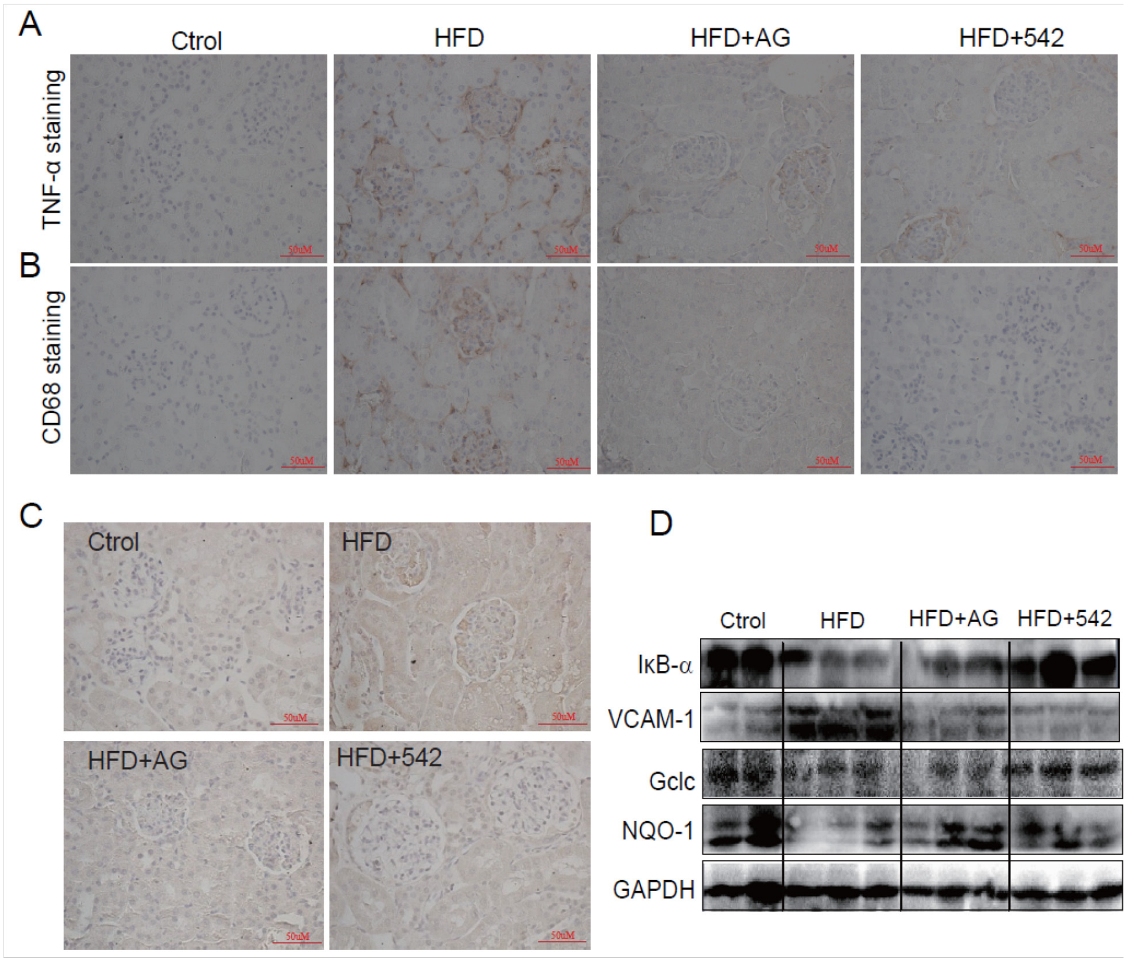

Figure S5: EGFR inhibition attenuates HFD-induced inflammation and ROS production in the kidneys of C57BL/6 mice. A-B. The administration of 542 for 2 months significantly reduced HFD-induced inflammation in the kidneys of obese mice, including the decreasing the expression of TNF-α (A) and macrophage infiltration, as shown through CD68 staining (B). **C.** 542 also decreased the HFD-induced oxidative stress, as shown in the results of 3-NT staining. **D.** Administration of 542 inhibited the degradation of IκB and production of VCAM-1 and increased the protein expression of anti-oxidants Gclc and NQO-1.

Table S1: Primer sequences for real-time quantitative PCR

Gene	Species	FW	RW
TNF-α	Rat	TACTCCCAGGTTCTCTTCAAGG	GGAGGCTGACTTTCTCCTGGTA
IL-6	Rat	GAGTTGTGCAATGGCAATTC	ACTCCAGAAGACCAGAGCAG
Gclc	Rat	TTGTTACTGAATGG CGGCGATGTT	GCGGGGGTGCTTGTTTATGG
HO-1	Rat	TCTATCGTGCTCGCATGAAC	CAGCTCCTCAAACAGCTCAA
NQO-1	Rat	ACTACGATCCGCCCCCAACTTCTG	CTTCGGCTCCCCTGTGATGTCGT
TGF-β	Rat	GCAACAACGCAATCTATGAC	CCTGTATTCCGTCTCCTT
CTGF	Rat	GCCTGTTCCAAGACCTGT	GGATGCACTTTTTGCCCTTCTTA
Collagen1	Rat	GACATCCCTGAAGTCAGCTGC	TCCCTTGGGTCCCTCGAC
β-actin	Rat	AAGTCCCTCACCCTCCCAAAAG	AAGCAATGCTGTCACCTTCCC
CTGF	Mouse	ACTATGATGCGAGCCAACTGC	TGTCCGGATGCACTTTTTGC
Collagen1	Mouse	TGGCCTTGGAGGAAACTTTG	CTTGGAAACCTTGTGGACCAG
TNF-α	Mouse	TGATCCGCGACGTGGAA	ACCGCCTGGAGTTCTGGAA
IL-6	Mouse	GAGGATACCACTCCCAACAGACC	AAGTGCATCATCGTTGTTCATACA
TGF-β	Mouse	TGACGTCACTGGAGTTGTACGG	GGTTCATGTCATGGATGGTGC
IL-1β	Mouse	GGAAGCACGGCAGCAGAATA	CCATCAGAGGCAAGGAGGAA
Nqo-1	Mouse	AGGGTTCGGTATTACGATCC	AGTACAATCAGGGCTCTTCTCG
Nrf2	Mouse	TTTTCCATTCCCGAATTACAGT	AGGAGATCGATGAGTAAAAATGGT
Gclc	Mouse	GCACGGCATCCTCCAGTTCCT	TCGGATGGTTGGGGTTTGTCC
HO-1	Mouse	GAGCAGAACCAGCCTGAACT	TTTGAACTTGGTGGGGCTGT
CollagenIV	Mouse	CAAGGACCGGTTTATTTGGC	ATTCCCTGCGAAGAACACAGC
β-actin	Mouse	CCGTGAAAAGATGACCCAGA	TACGACCAGAGGCATACAG
